# ‘No one cares about the animal like me.’ Indian veterinarians’ experiences of improving animal welfare through Continuing Professional Development

**DOI:** 10.1017/awf.2025.3

**Published:** 2025-02-03

**Authors:** Emma L Rayner, Ranjita Bastola, Sumanth Bedre, Andrew D Gibson, Luke Gamble, Jill RD MacKay

**Affiliations:** 1 Worldwide Veterinary Service, 4 Castle Street, Cranborne, Dorset, UK; 2 Worldwide Veterinary Service, International Training Centre, Gramya Bhavan, RDO Trust Building, Aruvankadu, The Nilgiris 643202, Tamil Nadu, India; 3 Royal (Dick) School of Veterinary Studies, The University of Edinburgh, Edinburgh, UK

**Keywords:** animal welfare, education, patient care, quality standards, skills-based learning, surgical outcomes

## Abstract

Veterinarians are custodians of animal welfare, ensuring practices remain current and effective in the face of the ever-changing demands of the profession. Continuing Professional Development (CPD) is essential for protecting animal welfare, however access to quality CPD is a challenge in many countries. India has one of the fastest growing veterinary industries globally, emphasising the importance of accessible CPD opportunities that are relevant to this setting. This study used focus groups to explore how Indian veterinarians identify relevant CPD, barriers they encounter, and their experiences with CPD. We describe three themes: (1) ‘career vs calling’, where veterinarians’ extrinsic and intrinsic motivational factors were identified, such as their desire to protect animal welfare; (2) being ‘willing to learn but can’t’, with context-specific barriers, such as accessing reliable CPD information; and (3) ‘real interactions matter’, where participants described how pedagogical design influenced their choices, e.g. being able to observe animal welfare improvements through practical teaching. We have three recommendations: firstly, to improve CPD learning opportunities informed by evidence-based methods, to meet knowledge and skills gaps such as the high demand for practically focused training; secondly, the development of a unified accreditation and quality assurance framework to assess content, relevance and delivery standards of available CPD options to veterinarians; and, lastly, improved support from employers to address current barriers and facilitate attendance. These findings contribute to the current knowledge gap of factors that influence Indian veterinarians’ experiences of attaining relevant, accessible CPD and makes suggestions to improve standards of veterinary care and, ultimately, patient welfare.

## Introduction

Veterinarians are custodians of animal welfare, and enhancing, protecting and securing the health and welfare of animals is the fundamental process of the veterinary profession (WSAVA 2024). To ensure veterinarians are able to carry out their roles effectively whilst keeping pace with ever-changing demands placed upon them (Wieland *et al.* 2021), they must be equipped with the necessary support and training. Continuing Professional Development (CPD) should provide such opportunities whatever their career stage, from building and enhancing basic skills acquired during undergraduate education through to specialist training. It is important that animal welfare science and pedagogical design interact in the creation of a learning opportunity to ensure that specialist animal welfare knowledge is delivered appropriately (MacKay 2020). Common veterinary CPD provisions are not without criticism, however, with the type of learning opportunity and the surrounding pedagogical framework often both lacking in forethought and consideration, resulting in poorer learning experiences (Wallace & May 2016). Delivering effective CPD requires knowing what veterinarians need to be competent at, ensuring it meets those needs and delivers education, and results in sustained changes in practices, demonstrated through formal assessment of CPD offerings (Gates *et al.* 2021). It is most commonly the veterinarians themselves who must seek out CPD, leading to calls for better frameworks and support to fulfil the obligation. There are a number of barriers veterinarians often face in the search for appropriate CPD, including finding time for meeting formal learning requirements, understanding which opportunities are of value to the individual concerned, and funding these opportunities (Dale *et al.* 2013; Gates *et al.* 2020).

In India, CPD participation is not mandated for veterinarians but attendance is encouraged by key stakeholders such as state veterinary councils and veterinary organisations (Indian Veterinary Association 2024). Delivering effective opportunities to veterinarians serving all work sectors is a key challenge; in India, for example, the scope of veterinary services is diverse, including a large focus on livestock health and welfare, and public health in a country where around 69% of the population live in a rural location (Rana 2017; Government of India 2024), to supporting the increasing pet ownership sector (Ankur 2023; Kalambi 2023) and the challenges of free-roaming dog population reduction and rabies elimination strategies (Gibson *et al.* 2018, 2022; Corfmat *et al.* 2023; Fielding *et al.* 2023). Furthermore, in general, veterinarians’ training requirements depend on their career stage; for newly qualified graduates, there is often a focus on achieving ‘day-one’ competences which, in some countries, have been identified as the core skills used routinely in veterinary practice (Welsh *et al.* 2009). Additionally, we know that mid-career veterinarians, having achieved clinical competency, likely face different challenges such as a change in career focus or a return from a career break, as well as more emotive issues such as compassion fatigue or ‘burn-out’ (Gates *et al.* 2021). At the later-career stage, where less attention has been focused traditionally, veterinarians may face challenges such as physical restrictions, or psychological factors such as self-esteem or a ‘sense of belonging’ (Gates *et al.* 2021). The CPD format should also reflect best practice in teaching, including opportunities for practical, ‘hands-on’ training and direct engagement with colleagues (Davis *et al.* 1999). Learning opportunities must therefore encompass both role diversity and career stage requirements and contextualise these for individuals’ circumstances, educational needs and interests (Dowling *et al.* 2018, 2019, 2020; Kiuru & Webster 2021). The outcomes of CPD attendance can be broad, encompassing knowledge and skills attainment, networking and community building, as well as achievement, validation and self-efficacy (Gates *et al.* 2020; Allen *et al.* 2021). However, these broad outcomes contribute, in part, to the current disagreement as to what constitutes *effective* CPD (Schostak *et al.* 2010; Gates *et al.* 2021; Kareskoski 2023). Despite this, it is clear that a major challenge is to ensure CPD outcomes uphold evidence-based medicine and promote behavioural change in practice (Gates *et al.* 2021). Veterinarians are required to attain and maintain professional competencies through the attendance of CPD opportunities relevant to their areas of expertise. The outcomes of said knowledge and skills attainment must further translate into improvements in workplace practices through sustained behavioural change, thereby upholding high standards of patient outcomes. A key aspect of ensuring that behavioural change is developed and maintained, is making sure that the educational opportunities are tailored for the specific context of the learners; and so it is important that we take time to understand how groups of veterinarians experience their learning to make improvements (MacKay & Pollock 2023).

It is not currently known how relevant CPD opportunities are for Indian veterinarians, whether CPD offerings satisfy individual requirements and expectations, and how these fit into the workplace context. In general, it is well documented that veterinarians have varying experiences with regard to factors such as their levels of engagement, motivational drivers and barriers to access (Moore *et al.* 2000; Delver 2008; Dale *et al.* 2013; Gates *et al.* 2020; Alafiatayo *et al.* 2022). There are also preferred methods of accessing CPD content (Moore *et al.* 2000; Delver 2008; Dale *et al.* 2013; Huntley *et al.* 2017; Gates *et al.* 2020; Alafiatayo *et al.* 2022) such as attendance programmes or remote access via a PC or mobile devices. In this study, we engaged groups of veterinarians attending training conducted by the Worldwide Veterinary Service (WVS) charity in India, to explore their experience of CPD. WVS runs regular canine surgical neutering courses and other CPD opportunities for domestic veterinarians in India and elsewhere. It has a strong focus on the provision of practical training opportunities to develop competencies and skills for veterinarians working in resource-limited settings and, concomitantly, improving animal welfare standards. In this study, we engaged a focus group approach to explore the veterinarians’ experiences engaging with CPD in India. We wanted to know: How do veterinarians in India identify and seek out relevant CPD experiences? What barriers exist to engaging with CPD? And what experiences do Indian veterinarians have engaging with practical CPD opportunities? We aim to inform future improvements in learning and development opportunities for veterinarians in India and other global settings.

## Materials and methods

The purpose of the study was to explore Indian national veterinarians’ experiences regarding CPD. A qualitative approach was adopted facilitating several focus groups conducted at an international veterinary charity’s surgical training facilities for veterinarians in India.

### Position of researchers

ER – a British-national, female veterinarian and AG – a British-national, male veterinarian, both employed in non-clinical, research-focused roles within the WVS charity. LG – a British-national, male veterinarian and CEO of the WVS charity. RB – a Nepalese-national, female veterinarian, and SB – an Indian-national, male veterinarian, employed in a clinical role for WVS in India at the time of the study. JM – Scottish-national female in a non-clinical role at R(D)SVS with a socio-constructivist epistemological position was the study supervisor. EB and JM were the analysis leads on this work.

### Ethical considerations

The project was approved by the University of Edinburgh Royal (Dick) School of Veterinary Studies Human Subject Ethical Review Committee in December 2023 (HERC_2023_166). All data were kept anonymous and in accordance with the UK General Data Protection Regulation (UK GDPR) and the Data Protection Act 2018. Participants were able to withdraw from the study up to the point of data analysis. No participants elected to withdraw.

### CPD provision

We selected the WVS charity’s canine surgical neutering training programme near Ooty, Tamil Nadu, India as our target programme. The 12-day, residential programme has been running for 14 years and is well established within the area. It focuses on equipping and improving national veterinarians with the surgical skills required for the neutering of free-roaming dogs. There is a strong focus on the provision of closely supervised, practical surgical neutering experience by nationally trained, Indian veterinary staff, with supplementary, didactic, lecture-based teaching covering a broad range of surgically related topics such as animal welfare, and the responsible use of antibiotics.

### Participant selection

Thirty veterinary surgeons attending the CPD programme were invited to attend one of a series of online focus groups. Participants were veterinary surgeons who had graduated from a national veterinary college in India and had varying professional backgrounds and years of working experience. Those who were interested in participating in the focus groups were provided with an information and consent sheet, and their verbal consent was attained prior to commencement. The focus groups were conducted within the constraints of a busy teaching facility resulting in challenges in conducting the sessions around the curriculum, staff availability, and the willingness of the participants to be involved in this additional activity in their free time. A limit of 30 participants was selected by the authors; this was predicted to provide sufficient opportunities to capture the breadth and diversity of participants’ views, whilst enabling the delivery of the focus groups within the practical constraints of this specific setting.

### Data collection

Between January and June 2024, nine focus groups were conducted with between three to four participants per session. We elected to use focus groups due to their structure of having more participants than researchers; these enabled participants to have ‘strength in numbers’ when talking to a researcher who may be perceived as having more power than them. We felt this was particularly useful in this context with a foreign researcher from the CPD-delivering charity asking the questions (Mackay & Wu 2024). In addition, this method enabled the delivery of the study within the context of a busy teaching facility which was not feasible for other methodologies such as individual interviews. A questionnaire approach was discounted due to its limitations in exploring individual experiences and perceptions in depth. The focus group schedule comprised a series of topics; exploring the benefits or advantages of CPD participation, barriers preventing access to attending CPD opportunities, the processes by which participants decide on the type of CPD, their views on the CPD format options, and how they feel that CPD can be improved for veterinarians in India. The groups were composed of those participants who had volunteered to be part of the study; two of the authors (ER, RB) moderated the sessions with ER taking the role of lead moderator in three sessions and RB in six. ER attended remotely using Microsoft® ‘Teams’ software (version 24231.512.3106.6573), whereas RB attended in person for all the focus groups. In addition, RB oversaw challenges occurring during the sessions such as re-phrasing questions due to language barriers, and asking additional questions, as necessary. Each focus group lasted between 30–40 min with the moderators ensuring that the maximum time-frame of 40 min was not exceeded. The focus groups were recorded, and transcripts generated, using Microsoft® ‘Teams’ software. ER sense-checked and edited transcripts prior to analysis.

### Data analysis

We elected to use a reflexive thematic analysis approach as per Braun and Clarke (2022). ER read through all focus group transcripts and discussed initial themes and approaches with JM. ER then went through an iterative coding process using Lumivero® ‘NVivo’ software (version 14:23.3). Developed themes were shared with JM who offered ideas for refinement, before ER described the three main themes from the data. There was limited opportunity for participant checking given the time between focus groups and analysis. RB informally reached out to a small number of participants to request feedback on their transcripts and resulting themes but did not receive any responses.

## Results

### Overview

The shared input of the participants provided rich and diverse accounts of their experiences in accessing and attending CPD within India. It was clear there was a strong drive for self-improvement, often strongly engrained within the values they held as veterinary professionals. Alongside this was a dedication and determination to succeed, often despite facing multiple, diverse barriers. The participants were clear on their expected outcomes from the CPD opportunities, which had a strong focus on ‘real life’ interactions, practical opportunities and interacting directly with peers and educators. Three themes emerged strongly from the data which helped us address our initial research questions: How do veterinarians in India identify and seek out relevant CPD experiences? What barriers exist to engaging with CPD? And what experiences do Indian veterinarians have engaging with practical CPD offerings? The three themes that emerged were: (1) ‘the career calling’, where veterinarians described their intrinsic and extrinsic motivation drives to improve their practice through CPD; (2) being ‘willing to learn but can’t’, where certain context-specific challenges such as difficulty seeking out reliable CPD information present barriers to the Indian veterinary context; and (3) ‘real interactions matter’, where participants highlighted the importance of pedagogical design in the experience of CPD and the value of interactivity. Each theme is described below and aims to communicate the diversity and richness of the data which underpin them.

### 1. ‘The career calling’

Participants discussed a range of perceived benefits to attending CPD, impacting them both at an individual level and in their professional delivery. These included internally driven, emotive goals such as personal satisfaction, enjoyment, and interest, as well as extrinsic, reward-driven incentives outwith the individual, including improvements in patient welfare or enhancements in their own working conditions such as pay or career advancement.

#### Personal improvement

Participants spoke of their desire to improve themselves on a personal level. They recognised the inherent value in their choice of profession, in what it stands for and the cause it serves. They wanted to ‘honour’ this status through self-improvement, not only simply fulfilling their responsibilities professionally but doing so with the level of respect and recognition they felt it deserved. This attained self-satisfaction on a personal level.

*“Like my personal opinion is I want to learn this for myself, it’s not just for the employer. So if I’ve joined this course, it’s for myself. And it’s basically enhancing my technical skills, technicalities and the subject. So we’re doctors. So I’m just doing justice to that. So you know, like that matters personally to me”* [Participant 3, FG 1].

*“By learning this course we can get professional satisfaction; like we choose this profession for a cause and we are doing it, and learning new things here that may give us human satisfaction like what we have studied, we are practicing here”* [Participant 3, FG 6].

#### Lack of confidence

Participants commonly experienced a lack of confidence in their capabilities, particularly in their practical skills, and addressing this was highlighted as a key, intrinsically motivating factor. There was a desire to be able to ‘stand on their own two feet’ without the need to ask for help and avoid the fear of appearing inadequate or unprofessional to their peers and their clients. Participants attributed this lack of confidence to a dearth of opportunities to practice and thereby improve their skills during their undergraduate training. This is a commonly reported concern for new graduates worldwide; they are inevitably expected to be competent in ‘day-one’ skills whilst having been afforded inadequate practical training opportunities, not only as students but also newly qualified veterinarians. It is well known that the transition from undergraduate to professional is challenging and can often result in a negative experience with detrimental consequences (Mellanby & Herrtage 2004; Rhind *et al.* 2011; Duijn *et al.* 2020; Vinten 2020).

*“Schools are pretty resource scarce these days. All of us don’t get a chance to put our hands on, you know, on the patient”* [Participant 1, FG 6].

*“I so wanted from very first year, I so wanted to learn, learn things by doing it by myself. I used to go to government veterinary hospital, private clinics, you know, so that I can learn new things about veterinary, you know, so I can practice myself, … and being engaged with animals and overall veterinary field. But I think I did never get the opportunity like that because no private clinics wants to take a risk. They don’t want with a beginner to approach their patients. They are afraid of it. They want they just want to make money out of the patient”* [Participant 2, FG 8].

#### Enhancing client communication

Participants believed that by improving their confidence in their abilities, they would correspondingly improve their professional skills such as verbal and non-verbal communication with clients, hoping to develop a relationship of trust, leading clients to return and/or recommend their services. They believed that these qualities were inherent and could not be ‘faked’ through false pretences. Therefore, gaining confidence was an extremely valuable CPD outcome.

*“Like if we are actually good with our profession, it conveys through our body language that we can actually assess things-…. so I mean it will be a good start to us and the confidence will actually come, will be shown through our work and the way we talk. Because a person who does not have good knowledge can’t actually pretend to be confident, or that pretence won’t last long and it won’t be real as well”* [Participant 2, FG 4].

#### Elevating animal welfare

Elevating animal welfare standards was a strong extrinsic benefit for professional improvement, where participants viewed their career as a vocation with an overarching ethical ‘stewardship’ to uphold and protect the health of these ‘voiceless patients’. They described their improved confidence and overall competency as having positive impacts on their patients’ welfare in multiple aspects of their work, such as improved surgical techniques, analgesia and responsible use of antibiotics. Regarding the latter, a wider duty outwith the individual patient was also considered a ‘One Health’ approach, and acknowledging the integral role that animal welfare plays in community health and well-being.

*“Because our overall ecosystem, it includes animals, animal health. And nowadays because of COVID also, One Health programme is on peak. If we all want to be healthy now, we should keep all the three wings, environmental health, animal health, human health, They should all go hand in hand”* [Participant 1, FG 8].

*“Definitely as a veterinarian, animal welfare definitely matters for me because I am the first priority who want to take care of the animal welfare and I feel that if I am not the person, no one with cares about the animal like that”* [Participant 3, FG 9].

#### Professional standing

Participants also saw extrinsic value to CPD opportunities, particularly practically orientated ones, involving maintaining a strong reputation with recognised professional ‘standing’ within the veterinary community, including employers. The conferred expertise and ability from these CPD offerings allow participants to progress faster in their career and improve earnings, and it was particularly noted in the context of practical CPD, although this was not always accessible to all.

*“So as all three of us have just passed out, we’re still at the start of our careers. So if we can do CPD at this stage, we would, it would help us climb the ladder much easier and much faster. And then you’re getting it at a later stage where we would have maybe the money to go and attend it. But we’ll be at a stage where we’re much, much older than what we are right now”* [Participant 1, FG 2].

*“And as for the career, like if you’re particularly talking about this organisation, it really matters. Like even on your resume if there are like the mentioning of CPD such as WVS, it has an impact on your salary bracket also”* [Participant 3, FG 1].

*“This* [‘WVS Canine Surgical Neutering Course’] *certificate that I’m going to get after this course is like it’s going to impact majorly on my salary and the way I’m being treated in the hospital as well. Like I’ll be given a lot of cases to spay and castrate and all that rather than the normal ops that I’ll be attending on to or general like a general position. From there I can get into an OT* [operating theatre] *or even assist the surgeons. So that upgradation in my career is that”* [Participant 2, FG 1].

### 2. ‘Willing to learn but can’t…’

Several significant barriers preventing vets attending CPD have been described and this sentiment was also reflected in our participants’ experiences. There was a strong desire from participants to learn, but several barriers were highlighted which inhibited or prevented this from happening which we grouped under three broad categories: being able to find relevant CPD opportunities; the limited number of places available; and the physical geography of India making some attendance impossible, and all three of these were addressed by one participant:

*“I think it’s really difficult to find course that you will have to know about now. When you get to know about it, the date is already passed or something like that and you wait for the next time. But you usually don’t get it because there is a lack of advertisement I feel. Because relevant information doesn’t come on time to different parts of the country”* [Participant 2, FG 8].

#### Finding CPD opportunities

There was an underlying sense of ‘comradery’ amongst participants to circulate information for new opportunities amongst themselves which highlighted an equity of access issue based on digital networking. ‘WhatsApp’ chat and ‘Instagram’ posts were commonly organised around undergraduate peer groups, as well as through workplace colleagues and university seniors. This approach should be considered somewhat distinct from the use of digital sites by educators to cultivate Professional Learning Networks (Trust *et al.* 2016) or ‘Communities of Practice’ formed by individuals as social systems to share knowledge and a common interest (Sharp *et al.* 2024); these networks often have multifaceted structures which serve a variety of functions, whereas often our participants were simply looking for a reliable hub of information, and social media was supplying that. This ‘enlightened self-interest’ approach ensured the reliable sharing of information for the prosperity of their peers, whilst also leading to self-benefit through their own knowledge gain. This provides an insight into participants’ regard of the community aspect of the veterinary profession. Community can be described as a “*feeling of fellowship with others, as a result of sharing common attitudes, interests, and goals*” (Gyles 2018) and taps into the entity of kinship towards their colleagues, to provide opportunities by sharing information, and unified by a common goal for self-improvement.

Whilst this approach had inherent benefits, the type of social media platform was perceived to be a potential limitation for some veterinarians who were not engaging with the more commonly used platforms; this concern was expressed with a concomitant desire to offer a remedy to ensure inclusivity. Older people’s attitudes towards use of digitalised technologies have been shown to be complex, influenced by their own characteristics as well as social and technology-related factors (Zhang 2023); for older generations of veterinarians, opportunities may be missed through the lack of engagement with digital formats of communication.

*“There are some peoples like about 30* [years old]*, they may be veterinarian, but they don’t have access* [to] *Instagram and all. We have to make them some WhatsApp group”* [Participant 2, FG 9].

Whilst participants relied upon their peer groups to share information themselves accordingly, there was a clear sense of ‘competitiveness’ for certain courses due to the limited number of places, particularly for those CPD opportunities that were practically focused. This highlighted a conflict in sharing information, with increased awareness on one hand, which may result in a reduced likelihood of attending a desired CPD session.

*“I mean, when the news came out, everyone was just barging in and, you know trying to get a seat over there. So yeah, there were limited seats. I couldn’t get into that”* [Participant 3, FG 1].

*“So especially for WVS this happens like I’ve been trying for two years as soon as it is there, and it’s just gone within one or two minutes”* [Participant 2, FG 1].

#### Limited availability

Another barrier that participants identified was achieving the right kind of teaching, such as practical teaching which was valued over didactic seminars and conferences. Even when a course was identified, many commented that the number of places available was often inadequate, limiting their chances of success. By the time they heard about a course they wanted to attend, places were inevitably already filled, evoking a sense of disappointment and frustration.

However, it was clear that many participants persevered in their attempts to gain a place on a CPD course of their choice that was over-subscribed, when they perceived the gains from attendance outweighed the barriers. The approach by learners to engage in a complex cognitive endeavour (described as a ‘deep approach’) has been negatively correlated with barriers and positively correlated with intrinsic, social and extrinsic motivating factors (Dale *et al.* 2010). This is akin to our participants’ persistence to engage in similar deep learning opportunities to improve their practical skills and subsequent confidence.

#### Perceptions of CPD providers

An interesting aspect to the participants’ evaluation of their CPD experience focused on their perceived relationship with the CPD provider, with reliability identified as a key factor. There was a feeling of uncertainty associated with some providers as to whether they could ‘trust’ them. This trust was key to deciding whether to invest themselves in a particular programme. Where a sense of trust had been established based on the participants’ previous experiences, this evoked a sense of providers having a ‘moral duty’ to supply future opportunities. Where CPD is regulated by an assessment and accreditation organisation, assurance is provided that a certain quality standard has been attained (CPD Certification Service 2020); whilst this does not currently exist within the Indian CPD framework, uncertainty can constitute a notable barrier for learners.

*“I feel there must be reliable organisation who can trust. We see a lot of programmes but we don’t know whether they are authentic or not….”* [Participant 1, FG 8].

“…*but in India there are no CPD programmes going on. So we need a lot of such things and we would expect like WVS to like come into all this because we have kind of a trust developed with this particular organisation based on this experience”* [Participant 2, FG 4].

#### Geographical constraints

Finally, another key barrier to CPD attendance was the geographical realities of India as a country. There were opportunities which were simply not available to participants due to their location or timing, and participants were keen for more opportunities to plug this gap. It was clear that CPD attendance was not commonly supported by employers, either financially or via the provision of paid leave, with the onus being on the veterinarian to organise. This forced participants to consolidate their decision-making process. It was clear that this ‘cost-benefit’ analysis appeared very much shaped by an individual’s own expectations of the gains versus the practical considerations they faced.

*“Location of the programme matters a lot because if person staying at Tamil Nadu cannot go to Kashmir, that matters a lot. We have to divide them somewhere because it should be conducted different parts of India. Then they will get some sort of knowledge*” [Participant 2, FG 9].

*“ …for just 10 days they were asking for ₹60,000 Indian rupees. So that’s a lot. That’s probably like more than one and a half month’s salary. So the fees is a problem for CPD we would say”* [Participant 3, FG 1].

### 3. ‘Real interactions matter….’

The opportunity to interact with people through ‘in-person’ attendance programmes was highly valued by many participants. When discussed in relation to practicing and improving their clinical skills, they were unanimous in their preference for practical opportunities compared to online alternatives. Whilst knowledge acquisition was more easily accessible via online options, practically orientated opportunities were considered very rare in India and, consequently, highly sought after.

#### Supervisory support

Participants described feeling reassured and supported by the presence of a supervisor whom they could immediately ask questions as they arose, and who could identify and correct any mistakes they made in a non-judgmental way.

*“This structure of CPD is the best thing I think because we are getting the training on hand. They are supervising us and they are letting us know our mistakes if we do something wrong”* [Participant 2, FG 2].

*“We see a lot of videos but hands-on makes our confidence level is high”* [Participant 3, FG 7].

Practical CPD led to an increase in motivation to continue when they could see tangible improvements in their abilities.

#### Real-life surgeries

They also described the experiential benefits of undertaking real-life surgeries, such as how different tissues or organs ‘felt’ and gauging tightness when placing sutures; these were also perceived as valuable outcomes linked to improved confidence in their abilities.

*“Actually in-person CPDs are good because we can have an easy access and we can touch and palpate a surgery. It’s not all about* [that] *we can ask the instructor about our doubts”* [Participant 2, FG 9].

This is in accordance with a review of the medical profession which demonstrated that activities that provide opportunities to practice skills, as well as other outcome-focused activities, often resulted in highly significant changes in practices as well as patient outcomes (Marinopoulos *et al.* 2007; Drexel *et al.* 2010). These output-focused measures of CPD attendance provide a meaningful gauge of genuine learning and professional improvement and are the current focus of CPD planners for improving strategies for effective CPD delivery worldwide.

#### Networking

Aside from the benefits of personal improvement in skill levels, many participants were eager to engage in professional networking which was considered a valuable opportunity to interact with other veterinarians, to learn from them and to share knowledge and experiences, as well as create new support networks.

*“When we go and meet different minds, new people and who are specialists in that field. Like there are vets coming up from abroad in different CPDs, there are different conferences, seminars, workshops, trainings going on and you know what is the latest going on in that field. And they are really helpful”* [Participant 1, FG 9].

These sentiments align with other evidence where value is placed on meeting with colleagues and sharing experiences (Wieland *et al.* 2021); this is particularly pertinent for professionals based in rural locations with fewer opportunities to engage with peers directly (Dowling *et al.* 2019; Kiuru & Webster 2021).

## Discussion

In this study, we utilised a focus group approach to facilitate the collection of data regarding the experiences of Indian veterinarians’ attainment of CPD. There were certain limitations associated with our study which are noteworthy. Demonstration of ‘trustworthiness’ was limited by the lack of opportunity for member checking of transcripts and feedback on the themes generated. Saturation was not actively ‘sought’ as a criterion for sufficient sample size, but the inclusion of 30 participants’ views provided a large data set whereby emerging themes were developed and re-analysed until there was no emergence of new themes. The study used as its data source the input from participants attending a canine surgical neutering training programme which focused on a specific skill set. The themes generated, whilst broad in their focus and perspective, likely offer potential for transferability to other contexts within the Indian veterinary CPD framework. For example, the motivations for veterinarians to seek CPD are likely shared due to inherent values and desires to improve both themselves and the care and welfare of their patients. Barriers to attendance, such as geographic location and limited opportunities, may also be applicable to other types of CPD offerings. Opportunities to attend specific training which facilitates practical skills improvement and peer interaction, is also likely shared amongst wider colleagues in a profession which is primarily skills-focused in its approach. Whilst limitations may make it harder to transfer or generalise from this study, they do not invalidate the findings and experiences of these participants.

Their experiences of engaging with CPD in some ways resembled the barriers and challenges faced by veterinarians in the UK, New Zealand, and elsewhere (Moore *et al.* 2000; Delver 2008; Dale *et al.* 2013; Gates *et al.* 2020) with factors such as geographic and financial constraints, lack of employer support and limited availability for attending CPD of their choosing. However, as may be expected, there were nuances of the experience of this group which require consideration when developing and evaluating CPD provision.

### Recommendations

We characterised three main themes: ‘career vs calling’; ‘willing to learn but can’t’; and ‘practical experiences matter’. The provision of ‘effective’ CPD for veterinarians in any country is a challenge, not least because what constitutes ‘effective’ has not been universally agreed upon (Kareskoski 2023). Here, we make three recommendations based on the Indian context which we think will improve CPD provision, and thus animal welfare, and can be learned from in other contexts. These are: recognising the importance of skills-focused training; adopting some form of quality assurance in CPD; and highlighting the need for industry and employer support.

### Skills-focused training

The challenges and preferences our Indian veterinarians faced as professionals share similarities to those in other countries (Moore *et al.* 2000; Delver 2008; Dale *et al.* 2013; Gates *et al.* 2020) and in other healthcare professions (Schostak *et al.* 2010; Dowling *et al.* 2018, 2019; Bwanga 2020) with regards to adequately equipping them to practice veterinary medicine from day one by providing sufficient practical opportunities in training. This highlights a key area for improvement in educational support.

With the current shift in global veterinary education towards a more competency-based approach, the delivery of CPD, and the methods of evaluating its outputs effectively, need to adapt alongside it. The key challenge in the evaluation of CPD in the veterinary and other healthcare sectors is demonstrating changes in practice at the patient level. Whilst traditional, input-focused delivery is often simple and cost effective and enables quantification of attendance (Friedman & Woodhead 2007), many have questioned the approach on the impact on practices. Our veterinary participants described how their experience with an interactive, practical training set-up empowered them with the confidence to take their skills forward into their working practices, and how this would improve patient welfare and care. Moreover, it was clear how much they valued this approach compared to the more traditional, didactic teaching approach (Wallace & May 2016). This approach is also preferred by medical physicians, with live, interactive activities that focus on knowledge and skill enhancement (Shah *et al.* 2017). Learning opportunities that focus on practical, interactive activities are shared across the national health and veterinary communities and are a key area for future address. These findings support the growing interest and movement towards an outcome-focused CPD approach in the global veterinary community, the premise of which seeks to evidentially measure outputs, specifically the impact on personal and professional development, and patient outcomes. This framework would support the expansion of the types of CPD opportunities, such as the one the WVS charity currently provides to Indian veterinarians, that encourage an interactive and a practical, skills-based approach; and with a focus on topics identified through evidence-driven methods. Whilst demonstrating long-term outcomes such as behaviour change and improved patient outcomes is challenging, there are models in medical practice that have demonstrated knowledge transfer into practice (Schostak *et al.* 2010; Wallace & May 2016). We know that obstacles to providing good animal welfare in veterinary settings, even in low- and middle-income countries (LMIC) are not necessarily knowledge-related, but rather about skills and context-specific barriers (Zaini *et al.* 2023). CPD being developed in such a way that it addresses these actual issues, rather than simply addressing the suspected issues, and engaging with the stakeholders throughout the CPD development process is key.

Implementation of such an approach requires an effective, regulatory framework. In those countries where such frameworks exist, approaches often differ widely (Bongers 2015; Wieland *et al.* 2021). Where CPD attendance is mandatory, this tends to be overseen by a Veterinary Council or Board, whereas in other countries, such as India, attendance is encouraged. In many LMIC, established veterinary CPD systems are frequently lacking due to funding, infrastructure and resource challenges (Giri *et al.* 2012), although, in the latter, there are a lack of published data. In Ethiopia, a comprehensive, evidence-driven approach has been adopted, headed by the Ethiopian Veterinary Association in collaboration with stakeholders and partners (Alafiatayo *et al.* 2022). A training needs assessment of the veterinary practitioners employed in the private and public sectors was conducted via a quantitative survey approach, the aim being to identify and prioritise key areas to deliver veterinary training. Subsequent outcomes would inform strategies to improve the skills, delivery, and governance of veterinary services across the country. A comprehensive initial assessment of needs, such as that demonstrated in Ethiopia, should be followed by policy-drafting incorporating regulatory and legal guidance, and training requirements, with an effective method for evaluation of impact. Our study has provided evidence of a shortfall in the training requirements for Indian national veterinarians in the area of practical skills improvement, and the need for a follow-on, comprehensive evaluation encompassing all work areas within the national veterinary sector.

### Quality assurance

The number of new course providers meeting increased demands for veterinary CPD has steadily increased in the last 20 years (Short *et al.* 2007), with digital learning abundant, and magnified by the COVID-19 pandemic (Boatright 2021); however, our veterinarians felt uncertainty regarding the quality of available CPD. Individual countries tend to work independently to implement their own accreditation system and CPD regulatory framework, resulting in wide variations in methodology and criteria. For example, in the US, the Registry of Approved Continuing Education (RACE) was established in 1997 and is a widely recognised method of accrediting veterinary education (Kareskoski 2023). They provide a list of approved programmes to veterinary professionals although it has limited use outside the US. The Federation of Veterinarians of Europe looked at nine countries and reported quality assurance to be provided in more than half, but in the majority it did not account for all training opportunities (Bongers 2015). The drivers for quality assurance provision vary, providers are not necessarily obliged to follow recommended standards; and the presence of a quality system does not imply mandatory CPD requirements and *vice versa.* Effective Communities of Practice (CoP) in veterinary education have been found to respond to community concerns, aligning to best-practice principles (Baillie *et al.* 2021), and given our participants shared resources and communications regarding CPD, a CoP approach may support the development of assured CPD provision within the country. This approach may also improve awareness of CPD opportunities available to veterinary professionals, which was a key shortfall described by our participants. A more unified approach to regulating and overseeing the quality of CPD opportunities globally should be considered as a future goal (Bongers 2015), alongside provision of officially approved course lists which allow easy access to veterinarians. Furthermore, CPD providers should engage with both animal welfare science and pedagogical approaches to ensure CPD is worthwhile (MacKay 2020) .

### Employer support

Whilst it ultimately remains the responsibility of the veterinarian to complete CPD, many employers, both in the private and public sector, and often in high income countries where CPD attendance is mandated, do offer varying subsidies such as provision of an annual monetary allowance and paid leave to facilitate engagement. The lack of employer support has been highlighted as a barrier to CPD attendance for veterinarians (Dale *et al.* 2013; Gates *et al.* 2020) and other professions (Davies & Preston 2002). Little appears to have been published as regards employers’ attitudes to CPD support, although a 2005 study on the business practices of a veterinary corporate organisations noted that relatively few respondents viewed CPD attendance as an opportunity to improve practice finances despite evidence showing the acquisition of new skills can support new revenue options (Volk *et al.* 2005). It is likely that certain employers may prioritise business considerations, such as profit and staff coverage, over employee CPD provision. However, where the benefits of employee lifelong training are identified by employers, this may encourage the facilitation of improved support. In many cases, a mismatch may well exist between the support graduates need from their employer and the level of support the employer is willing or able to supply; this may be compounded by unrealistic expectations placed on employees by the employer (Gates *et al.* 2021). A possible solution is to develop resources to facilitate communication around key topics such as job responsibilities, mentoring and CPD provision (Gates *et al.* 2021) and this should be aimed at the industry, stakeholders, and employers, as well as individual veterinarians, in order to combat this barrier. Inevitably, the delivery of an effective and sustainable approach to CPD opportunities must involve the co-operation of all key stakeholders, with acknowledgement of the benefits staff training bestows upon all parties.

## Animal welfare implications and conclusion

Supporting the continued education of veterinary professionals via the delivery of quality assured CPD, will provide individuals with the opportunities to improve their skills and their personal and professional development, thereby nurturing their commitment and passion for their chosen profession. Consequently, standards of patient care and welfare will ultimately be raised. Our participants clearly articulated the value they placed upon improving confidence in their abilities, and the concomitant benefits this imparted to the animals themselves. Whilst the experiences of our Indian veterinarians are not unique, they do highlight key areas for improvement in the access and delivery of CPD in their country. Harmonisation of an effective, international veterinary educational framework is desirable, which effectively demonstrates outcomes in patient care and welfare. The delivery of educational initiatives, including CPD, must be informed by each country’s requirements through evidence-based methods, such as the data presented in this paper. This will ensure that veterinary staff in all work sectors receive adequate support to realise their professional and personal goals.

## References

[r1] Alafiatayo R, Galipo E, Ekiri AB, Dineva M, Endacott I, Tesfaye T, Gellebo G, Awol F, Mijten E, Varga G and Cook AJC 2022 Training needs assessment of veterinary practitioners in Ethiopia. Tropical Animal Health and Production 54(1): 72. 10.1007/s11250-022-03075-0 35064854 PMC8783848

[r2] Allen LM, Palermo C, Armstrong E and Hay M 2021 Measuring impacts of continuing professional development (CPD): The development of the CPD impacts survey (CPDIS). Medical Teacher 43(6): 677–685. 10.1080/0142159X.2021.1887834 33635733

[r3] Ankur J 2023 *Future of the veterinary sector in India | The Conclusion!* https://www.linkedin.com/pulse/future-veterinary-sector-india-conclusion-ankur-jain (accessed 2 August 2024).

[r4] Baillie S, Rhind S, MacKay JRD, Murray L and Mossop L 2021 The VetEd Conference: Evolution of an Educational Community of Practice. *Journal of Veterinary Medical Education.* 10.3138/jvme-2020-0154 34097582

[r5] Boatright K 2021 The future of veterinary continuing education. *Today’s Veterinary Practice.* https://todaysveterinarypractice.com/personal-professionaldevelopment/the-future-of-veterinary-continuing-education/ (accessed 10 January 2025).

[r6] Bongers J 2015 *CPD in small animal medicine.* Federation of Veterinarians of Europe. chrome-extension://efaidnbmnnnibpcajpcglclefindmkaj/ https://fve.org/cms/wp-content/uploads/thesis_fve_quality_of_cpd_small_animal_medicine___jos_bongersfinal.pdf (accessed 10 January 2025).

[r7] Braun V and Clarke V 2022 Thematic Analysis. A Practical Guide. Sage Publications Ltd: London, UK.

[r8] Bwanga O 2020 Barriers to continuing professional development (CPD) in radiography: A review of literature from Africa. Health Professions Education 6(4): 472–480. 10.1016/j.hpe.2020.09.002

[r9] Corfmat J, Gibson AD, Mellanby RJ, Watson W, Appupillai M, Yale G, Gamble L and Mazeri S 2023 Community attitudes and perceptions towards free-roaming dogs in Goa, India. Journal of Applied Animal Welfare Science 26(4): 565–581. 10.1080/10888705.2021.2014839 35037536

[r10] CPD Certification Service 2020 *Veterinary & Animal | The CPD Certification Service.* https://cpduk.co.uk/industries/agriculture-veterinary/veterinary-animal (accessed 18 August 2024).

[r11] Dale VHM, Pierce SE and May SA 2010 The importance of cultivating a preference for complexity in veterinarians for effective lifelong learning. Journal of Veterinary Medical Education 37(2): 165–171. 10.3138/jvme.37.2.165 20576906

[r12] Dale VHM, Pierce SE and May SA 2013 Motivating factors and perceived barriers to participating in continuing professional development: a national survey of veterinary surgeons. Veterinary Record 173(10): 247–247. 10.1136/VR.101492 23980235

[r13] Davies R and Preston M 2002 An evaluation of the impact of continuing professional development on personal and professional lives. Journal of In-Service Education 28(2): 231–254. 10.1080/13674580200200181

[r14] Davis D, O’Brien MAT, Freemantle N, Wolf FM, Mazmanian P and Taylor-Vaisey A 1999 Impact of formal continuing medical education. Do conferences, workshops, rounds, and other traditional continuing education activities change physician behavior or health care outcomes? JAMA 282(9): 867–874. 10.1001/jama.282.9.867 10478694

[r15] Delver HA 2008 Continuing veterinary medical education needs and delivery preferences of Alberta veterinarians. Journal of Veterinary Medical Education 35(1): 129–137. 10.3138/jvme.35.1.129 18339967

[r16] Dowling S, Last J, Finnegan H, O’Connor K and Cullen W 2019 Does locally delivered small group continuing medical education (CME) meet the learning needs of rural general practitioners? Education for Primary Care 30(3): 145–151. 10.1080/14739879.2019.1573109 30747043

[r17] Dowling S, Last J, Finnegan H, O’Connor K and Cullen W 2020 What are the current ‘top five’ perceived educational needs of Irish general practitioners? Irish Journal of Medical Science 189(1): 381–388. 10.1007/s11845-019-02047-y 31190220

[r18] Dowling S, Last J, Finnigan H and Cullen W 2018 Continuing education for general practitioners working in rural practice: a review of the literature. Education for Primary Care 29(3): 151–165. 10.1080/14739879.2018.1450096 29623773

[r19] Drexel C, Merlo K, Basile JN, Watkins B, Whitfield B, Katz JM, Pine B and Sullivan T 2010 Highly interactive multi-session programs impact physician behavior on hypertension management: Outcomes of a new CME model. The Journal of Clinical Hypertension 13(2): 97–105. 10.1111/j.1751-7176.2010.00399.x 21272197 PMC8673395

[r20] Duijn C, Bok H, Ten Cate O and Kremer W 2020 Qualified but not yet fully competent: perceptions of recent veterinary graduates on their day-one skills. Veterinary Record 186(7): 216–216. 10.1136/VR.105329 31767696

[r21] Fielding HR, Fernandes KA, Amulya VR, Belgayer D, Misquita A, Kenny R, Gibson AD, Gamble L, Bronsvoort BM de C, Mellanby RJ and Mazeri S 2023 Capturing free-roaming dogs for sterilisation: A multi-site study in Goa, India. Preventive Veterinary Medicine 218: 105996. 10.1016/j.prevetmed.2023.105996 37595388

[r22] Friedman A and Woodhead S 2007 Approaches to CPD measurement research project. Accounting Education 16: 431–432. 10.1080/09639280701646646

[r23] Gates MC, McLachlan I, Butler S and Weston JF 2020 Practices, preferences, and opinions of New Zealand veterinarians towards continuing professional development. New Zealand Veterinary Journal 69(1): 27–37. 10.1080/00480169.2020.1803156 32781918

[r24] Gates MC, McLachlan I, Butler S and Weston JF 2021 Building veterinarians beyond veterinary school: Challenges and opportunities for continuing professional development in veterinary medicine 48(4): 383–400. 10.3138/JVME.2019-0148 34161200

[r25] Gibson AD, Mazeri S, Lohr F, Mayer D, Burdon Bailey JL, Wallace RM, Handel IG, Shervell K, de Bronsvoort BMC, Mellanby RJ and Gamble L 2018 One million dog vaccinations recorded on mHealth innovation used to direct teams in numerous rabies control campaigns. PLoS One 13(7). 10.1371/journal.pone.0200942 PMC6062050 30048469

[r26] Gibson AD, Yale G, Corfmat J, Appupillai M, Gigante CM, Lopes M, Betodkar U, Costa NC, Fernandes KA, Mathapati P, Suryawanshi PM, Otter N, Thomas G, Ohal P, Airikkala-Otter I, Lohr F, Rupprecht CE, King A, Sutton D, Deuzeman I, Li Y, Wallace RM, Mani RS, Gongal G, Handel IG, Bronsvoort M, Naik V, Desai S, Mazeri S, Gamble L and Mellanby RJ 2022 Elimination of human rabies in Goa, India through an integrated One Health approach. Nature Communications 13(1): 2788. 10.1038/s41467-022-30371-y PMC9120018 35589709

[r27] Giri K, Frankel N, Tulenko K, Puckett A, Bailey R and Ross H 2012 *Keeping up to date: Continuing professional development for health workers in developing countries.* https://www.semanticscholar.org/paper/Keeping-up-to-date%3A-Continuing-professional-for-in-Giri-Frankel/59a963669e584fbeb4588cdaed2d7abf7f7210d1 (accessed 5 September 2024).

[r28] Government of India 2024 *Data and resource.* https://censusindia.gov.in/census.website/ (accessed 19 September 2024).

[r29] Gyles C 2018 Veterinary medicine — profession and community. The Canadian Veterinary Journal 59(12): 1257. 30532282 PMC6237263

[r30] Huntley SJ, Dean RS and Brennan ML 2017 The awareness of the international veterinary profession of evidence-based veterinary medicine and preferred methods of training. Veterinary Sciences 4(1): 15. 10.3390/vetsci4010015 29056674 PMC5606622

[r31] Indian Veterinary Association 2024 *The Indian Veterinary Association- About us.* https://www.indianveterinaryassociation.in/about-us/ (accessed 13 September 2024).

[r32] Kalambi DS 2023 India’s pet healthcare industry: From ruff beginnings to pawsome prospects. *The Economic Times.* https://health.economictimes.indiatimes.com/news/industry/indias-pet-healthcare-industry-from-ruff-beginnings-to-pawsome-prospects/98843641 (accessed 13 September 2024).

[r33] Kareskoski M 2023 Accreditation in continuing veterinary education: development of an accreditation system and selection of accreditation criteria. Frontiers in Veterinary Science 10. 10.3389/fvets.2023.1181961 PMC10416105 37576832

[r34] Kiuru SP and Webster CS 2021 How might access to postgraduate medical education in regional and rural locations be best improved? A scoping review. Australian Journal of Rural Health 29(2): 236–244. 10.1111/ajr.12725 33848396

[r35] Mackay J and Wu S 2024 Educational research. In: Fogelberg K (ed) Educational Principles and Practice in Veterinary Medicine, First Edition, pp 415–418. Wiley Blackwell: London, UK. 10.1002/9781119852865.ch17

[r36] MacKay JR and Pollock K 2023 Human behaviour in veterinary care. *Animal Behaviour and Welfare Cases.* 10.1079/abwcases.2023.0021

[r37] MacKay JRD 2020 Discipline-based education research for animal welfare science. Frontiers in Veterinary Science 7. 10.3389/fvets.2020.00007 PMC6997439 32047758

[r38] Marinopoulos SS, Dorman T, Ratanawongsa N, Wilson LM, Ashar BH, Magaziner JL, Miller RG, Thomas PA, Prokopowicz GP, Qayyum R and Bass EB 2007 Effectiveness of continuing medical education. Evidence Report/Technology Assessment 149: 1–69. PMC4781050 17764217

[r39] Mellanby RJ and Herrtage ME 2004 Survey of mistakes made by recent veterinary graduates. Veterinary Record 155(24): 761–765. 10.1136/vr.155.24.761 15637999

[r40] Moore DA, Klingborg DJ, Brenner JS and Gotz AA 2000 Motivations for and barriers to engaging in continuing veterinary medical education. Journal of the American Veterinary Medical Association 217(7): 1001–1006. 10.2460/javma.2000.217.1001 11019705

[r41] Rana N 2017 Veterinary education in India: Shaping the future agenda with focus on veterinary public health education. The Indian Journal of Animal Sciences 87(9): 1052–1061.

[r42] Rhind SM, Baillie S, Kinnison T, Shaw DJ, Bell CE, Mellanby RJ, Hammond J, Hudson NPH, Whittington RE and Donnelly R 2011 The transition into veterinary practice: Opinions of recent graduates and final year students. BMC Medical Education 11(1): 1–10. 10.1186/1472-6920-11-64/TABLES/6 21939551 PMC3188471

[r43] Schostak J, Davis M, Hanson J, Schostak J, Brown T, Driscoll P, Starke I and Jenkins N 2010 ‘Effectiveness of continuing professional development’ project: A summary of findings. Medical Teacher 32(7): 586–592. 10.3109/0142159X.2010.489129 20653382

[r44] Shah MD, Goyal V, Singh V and Lele J 2017 Preferences and attitudes of physicians in India towards continuing medical education. Journal of European CME 6(1): 1332940. 10.1080/21614083.2017.1332940 29644133 PMC5843049

[r45] Sharp P, Baillie S, Parkes R, Janicke H, Kinnison T, Routh J, Muca E and Forrest N 2024 Setting up and running online communities of practice (CoPs) for veterinary educators. *Journal of Veterinary Medical Education.* 10.3138/jvme-2023-0042 39504210

[r46] Short N, Maddison J, Mantis P and Salmon G 2007 Veterinary e-CPD: a new model for providing online continuing professional development for the veterinary profession. Journal of Veterinary Medical Education 34(5): 689–694. 10.3138/jvme.34.5.689 18326783

[r47] Trust T, Krutka DG and Carpenter JP 2016 “Together we are better”: Professional learning networks for teachers. Computers & Education 102: 15–34. 10.1016/j.compedu.2016.06.007

[r48] Vinten C 2020 Making the transition to practice: building competence, confidence and trust. Veterinary Record 186(7): 213–215. 10.1136/VR.M576 32086415

[r49] Volk JO, Felsted KE, Cummings RF, Slocum JW, Cron WL, Ryan KG and Moosbrugger MC 2005 *Executive summary of the AVMA-Pfizer business practices study.* 10.2460/javma.2005.226.212 15706970

[r50] Wallace S and May SA 2016 Assessing and enhancing quality through outcomes-based continuing professional development (CPD): a review of current practice. Veterinary Record 179(20): 515–520. 10.1136/vr.103862 27856985 PMC5256232

[r51] Welsh P, Jones L, May S, Nunn P, Whittlestone K and Pead M 2009 Approaches to defining day-one competency: a framework for learning veterinary skills. 28(2): 771. 10.20506/rst.28.2.1921 20128489

[r52] Wieland B, Daborn C, Debnath N and Silva-Fletcher A 2021 Continuing professional development for veterinarians in a changing world. Revue Scientifique et Technique (International Office of Epizootics) 40(2): 555–566. 10.20506/rst.40.2.3245 34542094

[r53] WSAVA 2024 *Guidelines: Animal Welfare.* https://wsava.org/global-guidelines/animal-welfare-guidelines/ (accessed 27 September 2024).

[r54] Zaini SS, Phillips C, MacKay JRD and Langford F 2023 Perceptions of barriers to providing good cat care in Malaysian clinical practices. Animal Welfare 32: e70. 10.1017/awf.2023.91 38487432 PMC10936243

[r55] Zhang M 2023 Older people’s attitudes towards emerging technologies: A systematic literature review. Public Understanding of Science 32(8): 948–968. 10.1177/09636625231171677 37204075 PMC10631270

